# Semantic, phonologic, and verb fluency in Huntington’s disease

**DOI:** 10.1590/S1980-57642008DN10400009

**Published:** 2007

**Authors:** Mariana Jardim Azambuja, Monica Santoro Haddad, Marcia Radanovic, Egberto Reis Barbosa, Letícia Lessa Mansur

**Affiliations:** 1 MSc inExperimental Physiopathology.; 2 MD, MSc, Assistant-Physician Division of Neurology, Hospital das Clinicas, University of São Paulo School of Medicine, São Paulo, Brazil.; 3 MD, PhD Member of Behavioral and Cognitive Neurology Unit of the Department of Neurology, University of São Paulo School of Medicine, São Paulo, Brazil.; 4 MD, PhD, Associate-Professor, Department of Neurology, University of São Paulo School of Medicine, São Paulo, Brazil.; 5 PhD, Assistant-Professor, Department of Physiotherapy, Speech-Hearing-Language Pathology and Occupational Therapy, University of São Paulo School of Medicine

**Keywords:** Huntington’s disease, verbal fluency, neuropsychological tests, cognition, doença de Huntington, fluência verbal, testes neuropsicológicos, cognição

## Abstract

Verbal fluency tasks have been identified as important indicators of executive functioning impairment in patients with frontal lobe dysfunction. Although the usual evaluation of this ability considers phonologic and semantic criteria, there is some evidence that fluency of verbs would be more sensitive in disclosing frontostriatal physiopathology since frontal regions primarily mediate retrieval of verbs. Huntington’s disease usually affects these circuitries.

**Objective:**

To compare three types of verbal fluency task in the assessment of frontal-striatal dysfunction in HD subjects.

**Methods:**

We studied 26 Huntington’s disease subjects, divided into two subgroups: mild (11) and moderate (15) along with 26 normal volunteers matched for age, gender and schooling, for three types of verbal fluency: phonologic fluency (F-A-S), semantic fluency and fluency of verbs.

**Results:**

Huntington’s disease subjects showed a significant reduction in the number of words correctly generated in the three tasks when compared to the normal group. Both controls and Huntington’s disease subjects showed a similar pattern of decreasing task performance with the greatest number of words being generated by semantic elicitation followed by verbs and lastly phonologic criteria. We did not find greater production of verbs compared with F-A-S and semantic conditions. Moreover, the fluency of verbs distinguished only the moderate group from controls.

**Conclusion:**

Our results indicated that phonologic and semantic fluency can be used to evaluate executive functioning, proving more sensitive than verb fluency. However, it is important to point out that the diverse presentations of Huntington’s disease means that an extended sample is necessary for more consistent analysis of this issue.

## Abstract (pt)

As provas de fluência verbal têm sido consideradas como importantes indicadores de comprometimento executivo em pacientes com disfunção de lobo frontal. Embora a avaliação usual dessas habilidades seja baseada em critérios fonológicos e semânticos, existem evidências de que a fluência de verbos seria mais sensível para desvelar a fisiopatologia fronto-estriatal, já que regiões frontais são mediadoras primárias da recuperação de verbos e que a doença de Huntington afeta esses circuitos.

**Objetivo:**

Comparar três tipos de fluência verbal na avaliação da disfunção fronto-estriatal, em indivíduos com doença de Huntington.

**Métodos:**

Estudamos 26 sujeitos com doença de Huntington divididos em dois grupos: leve (11) e moderado (15), e 26 voluntários normais, emparelhados segundo idade, gênero e escolaridade, nas três condições de fluência: fonema inicial(F-A-S), fluência semântica (animais) e fluência de verbos.

**Resultados:**

Os indivíduos com doença de Huntington mostraram redução significante no número de palavras geradas corretamente nas três condições, quando comparados com o grupo normal. Em controles e na doença de Huntington, notamos comportamento semelhante: maior número de itens gerados por elicitação semântica, seguidos pelos critérios fluência de verbos e fonológico. Não encontramos alteração mais acentuada na produção de verbos, quando comparada com as condições F-A-S e semântica; a fluência de verbos distinguiu somente os doentes mais graves dos controles.

**Conclusão:**

Nossos resultados indicam a fluência fonológica e semântica podem ser utilizadas e são mais sensíveis do que a fluência de verbos para avaliar o funcionamento executivo. É importante pontuar, entretanto, que a diversidade de apresentação da doença de Huntington exige a ampliação da casuística para análise consistente do tema.

Verbal fluency tasks have been identified as important indicators of executive function impairment in patients with frontal lobe dysfunction.^1^ Thus, various studies have indicated poor performances on phonologic and semantic fluency tests in individuals with Huntington’s disease (HD).^1-6^ The loss in verbal fluency appears early in the course of HD and worsens as the disease progresses.^7^

Rosser and Hodges^1^ compared patients with Alzheimer’s disease (AD), Progressive Supranuclear Paralysis (PSP) and Huntington disease (HD) in semantic verbal fluency and phonological tasks. They suggested that the poor performance in patients with frontal-executive dysfunction is related to problems in initiating mechanisms to recuperate information secondary to the rupture of the frontal-striatal circuit. The same pattern was perceived in other studies,^6-8^ one of them using priming tasks.^9^ According to these authors, the poor performance reflects a difficulty in generating strategies that enable retrieval of the stored information.

Although the usual evaluation of this ability is performed using the letter and semantic criteria, there is some evidence^10,11^ that action fluency would be more sensitive to disclose fronto-striatal physiopathology since verb retrieval is primarily mediated by frontal regions.

Indications of performance dissociation in tasks to generate nouns and verbs were proposed by Piatt,^10^ who verified that verb fluency is disproportionately prejudiced in patients with Parkinson’s dementia. Cappa^11^ also identified an important role of the frontal lobe in action naming.

To date, the performance of patients with HD in verb fluency has been little investigated. In patients with HD and dementia, worse results in the generation of verbs than nouns were found.^12^

Our objective was to compare three types of verbal fluency task in the assessment of frontostriatal dysfunction in HD subjects.

## Methods

We studied 26 patients with HD and 26 normal volunteers in three types of verbal fluency: initial phoneme fluency (F-A-S), semantic fluency (animals) and fluency of verbs. The HD group was divided into two subgroups – mild (11) and moderate (15) patients – according to functional capacity, measured by the Unified Huntington Disease Rating Scale.^13^ The criteria of inclusion for the control group were: absence of cognitive complaints, absence of previous neurological or psychiatric disease, and normal neurological examination.

Each participant performed the Mini Mental State Examination (MMSE) and three verbal fluency tasks: phonemic, semantic and fluency of verbs. For phonologic fluency, three trials were performed (with the phonemes F, A, S). For semantic and fluency of verbs, subjects were asked to generate as many words as possible for animal and verb categories, respectively. For each category 60 seconds were allowed. To better characterize the alterations encountered, the errors on the fluency tests were classified into perseverations (repetition of an item that has already been mentioned or a word with a different suffix), intrusions (the inclusion of an item from another category or, in the case of phonological, emitting a word that begins with another phonological) or others (inappropriate answers that cannot be classified as described above).^1^ Patients and controls signed a Term of Informed Consent and the research was approved by the Ethics Committee of Hospital das Clínicas da FMUSP under the number 075/03.

### Statistical analysis

The results obtained for each task were compared for intergroups using ANOVA with the Bonferroni post-test. Analysis of Covariance (ANCOVA) was performed to control for differences in schooling among groups for all variables of interest. The significance value adopted was 5%.

## Results

Age did not differ among the three groups. Educational level was statistically different between mild and moderate HD patients. The control group had higher scores on the Mini Mental Status Examination (Table 1). HD patients of the two groups showed a significant reduction in the number of words generated in the FAS and animal fluency tasks compared to normal. Controls and moderate HD groups differed for verbal fluency (Table 2). For both groups, we noted similar performance patterns where the greatest number of words was generated in the animal category, followed by verbs and phonemic. Relative to the number of “perseverations”, there was a difference between control and moderate groups in the animal fluency task, whereas considering “other type of errors” the controls differed from moderate, and the mild differed from the moderate group in verb fluency (Table 3). Additional analysis of Covariance (ANCOVA) was performed to control the influence of schooling on all variables of interest (Table 4). The level of schooling only influenced the MMSE scores.

**Table 1:** Age, education and MMSE scores for controls and HD patients.

Variable	Controls (26)	Mild HD (11)	Moderate HD (15)	P	Intergroup difference
Age	47.9 (10.5)	45.7 (10.9)	49.5 (11.3)	0.678	-
Schooling	8.85 (4.45)	11.4 (3.2)	7 (3.67)	0.029	Mild & moderate HD
MMSE	28 (1.4)	24.7 (1.6)	20.1 (3.8)	< 0.0001	All groups differ

HD: Huntington's disease; MMSE: Mini Mental State Examination; all values are expressed as mean (Standard Deviation).

**Table 2:** Total number of words generated in verbal fluency tasks for controls and HD patients.

Variable	Controls (26)	Mild HD (11)	Moderate HD (15)	P	Intergroup difference
FAS	10.6 (2.8)	6.2 (2.7)	3.3 (2)	< 0.0001	All groups differ
Animal fluency	15.8 (4.9)	11.3 (3.8)	6.7 (2.9)	< 0.0001	All groups differ
Verb fluency	12.2 (5.3)	8.3 (3.5)	4.7 (4.4)	< 0.0001	Controls & moderate HD

HD: Huntington's disease; all values are expressed as mean (Standard Deviation).

**Table 3:** Performance of HD patients and controls relative to the number of perseverations, intrusions and other errors in the fluency tasks.

	Controls (26)	Mild HD (11)	Moderate HD (15)	p	Intergroup difference
**Perseverations**					
FAS Animal fluency Verb fluency	0.8 (0.7) 0.4 (0.7) 0.5 (0.8)	1 (0.9) 0.5 (0.9) 0.6 (0.8)	0.7 (0.6) 1.4 (1.4) 0.4 (0.9)	0.574 0.017 0.885	- Controls & moderate HD -
**Intrusions**					
FAS Animal fluency Verb fluency	0 (0) 0 (0) 0.1 (0.3)	0.1 (0.1) 0.1 (0.3) 0 (0)	0.1 (0.2) 0 (0) 0.1 (0.3)	0.132 0.156 0.661	- - -
**Other**					
FAS Animal fluency Verb fluency	0.3 (0.4) 0 (0) 0.2 (0.4)	0.2 (0.3) 0 (0) 0.2 (0.4)	0.8 (1.2) 0.1 (0.3) 0.9 (1)	0.121 0.078 0.004	- - Controls & moderate HD Mild & moderate HD

HD: Huntington's disease; all values are expressed as mean (Standard Deviation).

**Table 4:** Analysis of Covariance (ANCOVA) results, controlling for schooling in all variables of interest.

Dependent variable	Significance (p)
MMSE	0.002
FAS	0.300
Animal fluency	0.135
Verb fluency	0.230
Perseveration FAS	0.135
Intrusions FAS	0.212
Other errors FAS	0.156
Perseveration animal fluency	0.806
Intrusions animal fluency	0.442
Other errors animal fluency	0.471
Perseveration verb fluency	0.572
Intrusions verb fluency	0.423
Other errors verb fluency	0.871

## Discussion

The verbal fluency tasks are traditional measures of executive function, demanding the skill to mentally manipulate and co-ordinate a large quantity of diverse information, in order to recall elements of a given category.^10^ They also depend on the integrity of semantic and phonological memory. Since Huntington’s Disease is characterized by a functional decline in the fronto-subcortical system, there is expected impairment on fluency tasks – especially fluency of verbs, considered by some to be more sensitive to detect fronto-striatal damage.

In this study HD patients with varying degrees of disease evolution performed worse than controls on the three proposed tasks (semantic, phonologic and fluency of verbs). Nevertheless, both groups (HD and controls) behaved similar in terms of performance patterns, producing more words in the semantic than in the phonologic task.

The difficulties in verbal fluency tasks experienced by individuals with HD have already been amply described in the literature.^1,3,10,14^ Akin to the present study, Hodges^15^ and Rosser and Hodges^1^ found similar results in patients with HD and controls, with greater recall of words in semantic fluency than in phonologic fluency. Although the two tasks demand the same processes of unleashing and monitoring, controlled by a central executive, this difference appears to reflect the nature of the semantic representations as opposed to the letter instances or specificity of clues used for recall in each of the tasks. There are indications that the representation of the semantic system is organized into categories, leading to easier recall of semantically related items compared with other conditions. Phonologic fluency depends on the phonologic level of the word representation, without reference to a meaning and as a result, with a slower activation velocity.^1^

The poor results in patients with HD on verbal fluency tasks has been attributed to an alteration of the frontostriatal circuits,^10^ which control aspects of the executive function and include attention, information recovery, and operational memory. Majority of authors believe that in HD this reflects the difficulty in generating strategies to search for information rather than a compromise of semantic memory^1,11^. The fact that HD patients present better results in semantic fluency than in phonologic, an aspect found in this work and reports by other authors,^1,5^ advocates the view that good performance on semantic fluency depends more on preservation of semantic storage than phonologic and fluency of verb tasks. Furthermore, Rosser and Hodges^1^ found an opposite pattern in patients with Alzheimer’s disease who had worse results in semantic than in phonologic fluency compared to patients with HD and Progressive Supranuclear Paralysis, a dissociation which reinforces these findings. The qualitative analysis of the types of errors also helps in identifying the nature of the difficulties encountered. We verified statistical difference in the number of perseverations, in the task of animal fluency, between controls and moderate HD group while other type of errors (e.g. proper names, non-words, superordinate categories), also showed differences between controls and the moderate HD group as well as between mild and moderate HD groups. Our results differed from Pillon^16^ and Péran^12^ who did not find a different frequency of intrusions or perseverative errors in retrieval tasks of verbal material and generation of nouns and verbs, between HD and controls. Our findings suggest the necessity of additional investigation to verify the reasons for the particular performance observed in verbal fluency tasks and ascertain whether the *errors* are related to a specific loss in the elaboration of strategies to search for information, or self-monitoring, or inhibition of responses, all characteristics of executive function loss.

In our study, besides the traditional measures (semantic and phonologic fluency), we also performed a verb fluency task, not usually explored but indicated as sensitive for frontostriatal physiopathology.^10^ Unexpectedly, we did not find a significantly greater impairment in the production of verbs when compared to other fluency. For the two HD groups, performance on this verb task was lower than for semantic fluency but higher than for letter fluency, a pattern which repeated for the control group.

To our knowledge, no studies have yet evaluated verb recall and compared this with semantic and phonologic fluency in HD patients. Peran^12^ investigated concrete noun generation and actions in 26 patients with HD, 17 without dementia and 9 with. This task is differs to that of fluency, as verbal stimuli are presented (nouns or verbs) to be paired with semantically related items of the same category (nouns with nouns and verbs with verbs) or of the opposite category (nouns with verbs and verbs with nouns). However, the task shares some similar characteristics to the fluency task, due to the need for recall and transference between different categories. In this study, the HD group presented worse results than the controls in the four tasks. However, only the group with dementia displayed a significant difference between the generation of verbs and nouns, with worse results for the two tasks where generation of verbs was required. Piatt^10^ also verified that verb fluency differentiated patients with Parkinson and dementia from control individuals. Damasio and Tranel^17^ found difficulties in generation of verbs but not in nouns, in patients with a frontal lesion, while the opposite pattern was verified in patients with a posterior lesion. Cappa^11^ observed similar dissociations between patients with Alzheimer’s and fronto-temporal dementia. Our study found worse results in verbs than in semantic and phonologic fluency. However, the fact that none of these studies analyzed the three tests jointly prevents comparison with our results.

One of the reasons for better results in fluency of verbs than in the phonologic task could be related to the large variety of possible existing verbs (verbs which represent actions, feelings, states). At least a proportion of the verbs can be recovered using semantic strategies, which would facilitate the results in this task compared to phonologic fluency. Supposing that the verb fluency task is supported in the two networks (attentional and semantic), we could assume that both phonologic and semantic fluency would be sensitive to detect executive function problems in the early phases of HD. With the evolution of the disease, fluency of verbs would indicate the intensification of these difficulties.

In the future, a broader qualitative analysis investigating different types of verbs could further understanding of this task.

In our sample, schooling influenced the MMSE yet did not significantly interfere in fluency performance. Both phonologic and semantic fluency remained the most sensitive methods to detect fluency difficulties related to executive functioning. Nonetheless, it is important to point out that HD patients represent a heterogeneous group in terms of presentation and a larger sample is necessary to confirm these results.

## References

[1] Rosser A, Hodges JR (1994). Initial letter and semantic category fluency in Alzheimer's
disease, Huntington's disease, and progressive supranuclear
palsy. J Neurol Neurosurg Psychiatry.

[2] Bayles KA, Tomoeda CK (1983). Confrontation naming impairment in dementia. Brain Lang.

[3] Tröster AI, Salmon DP, McCullough D, Butters N (1989). A Comparison of the category fluency deficits associated with
Alzheimer's and Huntington's disease. Brain Lang.

[4] Troyer AK, Moscovitch M, Winocur G (1997). Clustering and switching as two components of verbal fluency:
evidence from younger and older healthy adults. Neuropsychology.

[5] Ho AK, Sahakian BJ, Robbins TW, Barker RA, Rosser AE, Hodges JR (2002). Verbal fluency in Huntington's disease: a longitudinal analysis
of phonemic and semantic cluster and switching. Neuropsychologia.

[6] Arango-Lasprilla JC, Rogers H, Lengenfelder J, Deluca J, Moreno S, Lopera F (2006). Cortical and subcortical diseases: do true neuropsychological
differences exist?. Arch Clin Neuropsychol.

[7] Butters N, Wolfe J, Granholm E, Martone M (1986). An assessment of verbal recall, recognition and fluency abilities
in patients with Huntington's disease. Cortex.

[8] Hodges JR, Salmon DP, Butters N (1991). The nature of the naming deficit in Alzheimer's and Huntington's
disease. Brain.

[9] Smith S, Butters N, White R, Lyon L, Granholm (1988). Priming semantic relations in patients with Huntington's
Disease. Brain Lang.

[10] Piatt AL, Fieldes JA, Paolo AM, Tröster AI (1999). Action (verb naming) fluency as an executive function measure:
convergent and divergent evidence of validity. Neuropsychologia.

[11] Cappa SF, Binetti G, Pezzini MA, Padovani A, Rozzini L, Trabucchi M (1998). Object and action naming in Alzheimer's disease and
frontotemporal dementia. Neurology.

[12] Péran P, Démonet JF, Pernet C, Cardebat D (2003). Verb and noun generation tasks in Huntington's
disease. Mov Disord.

[13] Huntington Study Group (1996). Unified Huntington's disease rating scale: reliability and
consistency. Mov Disord.

[14] Butters N, Granholm E, Salmon DP, Grant I, Wolfe J (1987). Episodic and semantic memory: A comparison of amnesic and
demented patientes. J Clin Exp Neuropsychol.

[15] Hodges JR, Salmon DP, Butters N (1990). Differential impairment of semantic and episodic memory in
Alzheimer's and Huntington's disease: a controlled prospective
study. J Neurol Neurosurg Psychiatry.

[16] Pillon B, Deweer B, Agid Y, Dubois B (1993). Explicit memory in Alzheimer's, Huntington's, and Parkinson's
Diseases. Arch Neurol.

[17] Damasio AR, Tranel D (1993). Nouns and verbs are retrieved with differentially distributes
neural systems. Proc Natl Acad Sci USA.

